# Tranexamic acid for the prevention of postpartum bleeding: Protocol for a systematic review and individual patient data meta-analysis

**DOI:** 10.12688/gatesopenres.13747.2

**Published:** 2023-05-12

**Authors:** The Anti-fibrinolytics Trialists Collaborators – Obstetric Trialists Group, Katharine Ker, Haleema Shakur-Still, Loïc Sentilhes, Luis D. Pacheco, George Saade, Catherine Deneux-Tharaux, Amy Brenner, Raoul Mansukhani, François-Xavier Ageron, Danielle Prowse, Rizwana Chaudhri, Oladapo Olayemi, Ian Roberts

**Affiliations:** 1 CTU Global Health Trials Group, London School of Hygiene & Tropical Medicine, London, WC1E 7HT, UK; 2 Department of Obstetrics & Gynecology, Bordeaux University Hospital, University of Bordeaux, Bordeaux, France; 3 Division of Maternal-Fetal Medicine, Department of Obstetrics & Gynecology, The University of Texas Medical Branch at Galveston, Galveston, Texas, USA; 4 Department of Obstetrics and Gynecology, Eastern Virginia Medical School, Norfolk, Virginia, USA; 5 INSERM U1153, Obstetrical, Perinatal and Pediatric Epidemiology Research Team, Center for Epidemiology & Statistics Sorbonne Paris Cité, Paris Descartes University, Paris, France; 6 Department of Emergency Medicine, Lausanne University Hospital, University of Lausanne, Lausanne, Switzerland; 7 Rawalpindi Medical College, Rawalpindi, Pakistan; 8 University of Ibadan College of Medicine, Ibadan, Nigeria

**Keywords:** Anti-fibrinolytics, Tranexamic acid, childbirth, postpartum haemorrhage, meta-analysis

## Abstract

Background: Tranexamic acid (TXA) reduces the risk of death and is recommended as a treatment for women with severe postpartum bleeding. There is hope that giving TXA shortly before or immediately after birth could prevent postpartum bleeding. Extending the use of TXA to prevent harmful postpartum bleeding could improve outcomes for millions of women; however we must carefully consider the balance of benefits and potential harms. This article describes the protocol for a systematic review and individual patient data (IPD) meta-analysis to assess the effectiveness and safety of TXA for preventing postpartum bleeding in all women giving birth, and to explore how the effects vary by underlying risk and other patient characteristics.

Methods: We will search for prospectively registered, randomised controlled trials involving 500 patients or more assessing the effects of TXA in women giving birth. Two authors will extract data and assess risk of bias. IPD data will be sought from eligible trials. Primary outcomes will be life-threatening bleeding and thromboembolic events. We will use a one-stage model to analyse the data. Subgroup analyses will be conducted to explore whether the effectiveness and safety of TXA varies by underlying risk, type birth, maternal haemoglobin (Hb), and timing of TXA. This protocol is registered on PROSPERO (CRD42022345775).

Conclusions: This systematic review and IPD meta-analysis will address important clinical questions about the effectiveness and safety of the use of TXA for the prevention of postpartum bleeding that cannot be answered reliably using aggregate data and will inform the decision of who to treat.

PROSPERO registration: CRD42022345775

Keywords

Anti-fibrinolytics; Tranexamic acid; childbirth; postpartum haemorrhage; meta-analysis

## Introduction

### Description of the condition

All women bleed after childbirth. For most women, the bleeding is modest and well tolerated but for some women, it can be serious and life-threatening. Indeed, with about 70,000 deaths every year world-wide ^1^, bleeding after childbirth is a leading cause of maternal death. Almost all (99%) maternal deaths from bleeding after childbirth are in low- and middle-income countries ^2^. In sub-Saharan Africa and south Asia, one woman dies from bleeding for every 1,000 births, while in high-income countries, there is less than one bleeding death for every 100,000 births ^3^. Regardless of setting, most deaths are on the day of the birth, many within the first few hours ^4^.

Women who survive severe bleeding can suffer significant physical and psychological morbidity which limits their well-being as well as their ability to breast-feed and care for their baby ^5^. Many women undergo urgent, invasive procedures such as hysterectomy, intrauterine balloon insertion or arterial ligation in an effort to stop the bleeding. Blood loss can also cause or worsen maternal anaemia, resulting in fatigue and an increased risk of postpartum depression ^6^. Severe bleeding is a frightening experience with severe psychological consequences such as post-traumatic stress disorder ^7, 8^.

### Description of the intervention

Tranexamic acid (TXA) reduces bleeding by inhibiting the breakdown of fibrin blood clots ^9^. Since its invention in the early 1960s, TXA has been used for heavy menstrual bleeding and to reduce surgical blood loss. A systematic review of clinical trials of TXA in surgery showed that it reduces the risk of blood transfusion by about one third ^10^. More recently, the POISE-3 trial with nearly 10,000 high-risk patients undergoing non-cardiac surgery found that TXA given at the start and at the end of surgery reduced the chance of life-threatening or serious bleeding by about one quarter ^11^. The CRASH-2 trial involving 20,211 bleeding trauma patients showed that early TXA treatment reduced bleeding deaths by one third ^12^. The WOMAN trial of TXA in severe postpartum bleeding also showed that early TXA use reduced bleeding deaths by one third ^4^. In both the CRASH-2 and WOMAN trials, early treatment was most effective, raising the possibility that TXA given before or immediately after birth could prevent severe bleeding. This would be particularly beneficial for women with anaemia who have a high risk of severe bleeding and for whom even modest bleeding can be harmful ^13^. The World Health Organization (WHO) currently recommends TXA treatment for all women with severe bleeding after childbirth ^14^. However, extending TXA use in women shortly before or after birth to prevent harmful bleeding, could improve outcomes for millions of women. 

### Why this research is needed

Although expanding the use of TXA to include the prevention postpartum bleeding could have major benefits, we must carefully consider the balance of benefits and potential harms. Pregnant women have an increased risk of arterial and venous thrombosis due to the increased propensity of the blood to clot and pressure from the expanding womb. Compared to women who are not pregnant, their risk of thromboembolism is five times higher, rising to 20 times higher in the postpartum period ^15^. Although there is no evidence from randomised trials that TXA increases the risk of thrombosis, because thrombosis is rare, the estimates are imprecise, and a small increased risk cannot be ruled out. Since the risks of postpartum bleeding and thrombosis vary between women, even if TXA is shown to prevent postpartum bleeding, for some women the potential harm may exceed the benefit. The challenge is to identify women for whom the benefits outweigh any harms.

**Figure 1.**
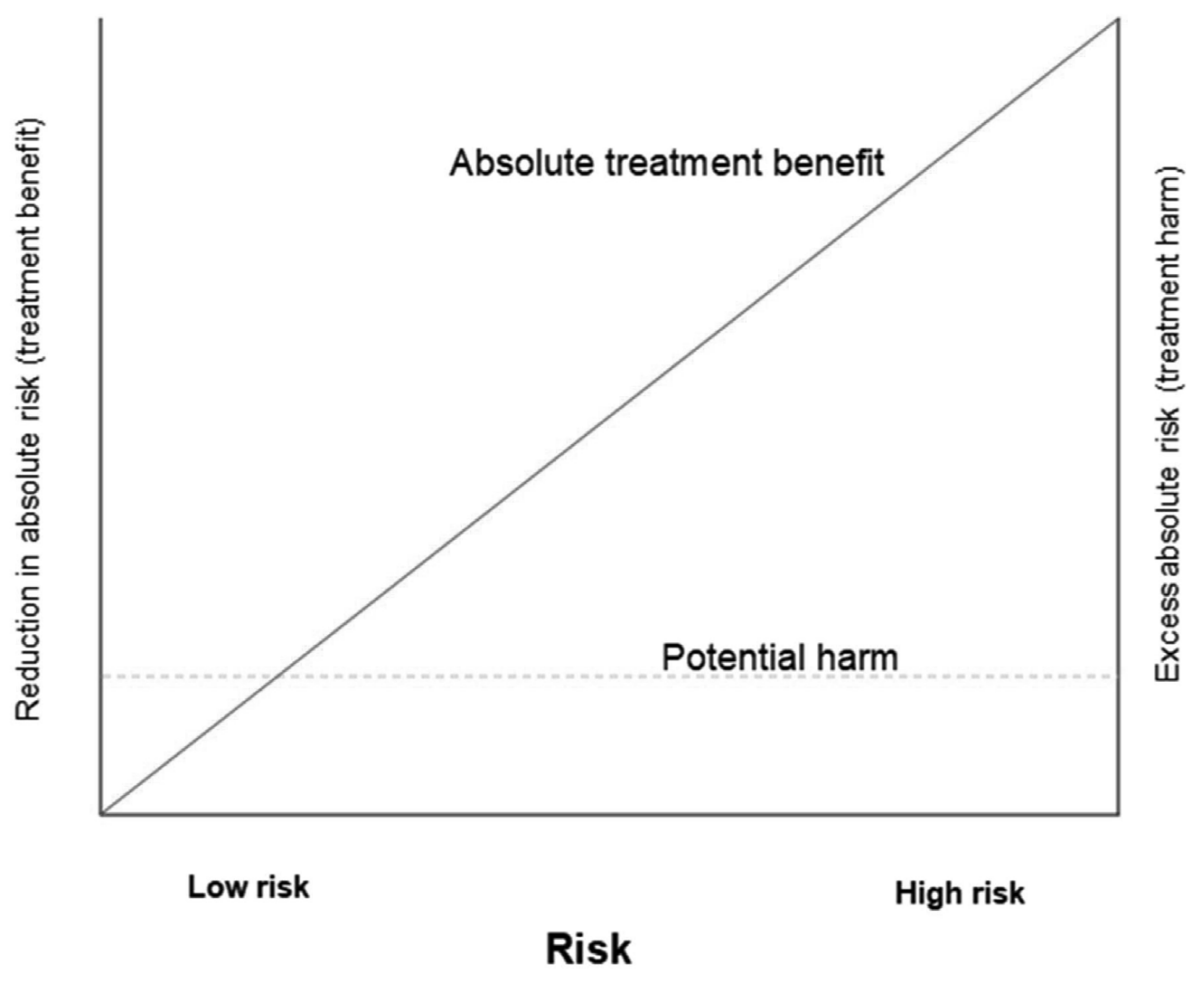
How the potential benefit and harm of a treatment varies by baseline risk. Adapted from Glasziou and Irwig ^16^.

Figure 1 shows how the benefits and harms of a treatment might vary by underlying risk. The absolute benefit from treatment increases with increasing underlying risk (as shown by the solid diagonal line). In trauma and postpartum haemorrhage, early TXA reduces the risk of death from bleeding by about one third regardless of underlying risk. For a patient with a 30% underlying risk of death, TXA would reduce the risk by one third to 20%. For a patient with a 3% underlying risk of death, TXA would also reduce the risk by one third to 2%. Both patients have the same proportional reduction in risk ( *i.e.* one third), but the absolute benefit varies substantially (30%-20%=10% versus 3%-2%=1%) because their underlying risks are different. However, the risk of harm is often constant across different levels of underlying risk (as shown by the dashed, horizontal line). For this reason, treatment benefits are more likely to exceed any harms in high-risk patients. We need to be more cautious about offering interventions to patients at low underlying risk since the balance of risks and benefits is more uncertain in these patients than in patients with high underlying risk. 

To identify women who would derive a net benefit from receiving TXA to prevent postpartum bleeding, we need reliable estimates of the effects of TXA on bleeding (potential benefit) and thrombosis (potential harm), and to assess whether and how these effects vary by underlying risk.

It is also important to consider potential neonatal effects associated with maternal TXA administration. TXA crosses the placenta and is present in umbilical cord blood at a similar concentration as in the maternal blood ^17, 18^. There is no evidence from randomised trials for neonatal harm following exposure via placental transfer, although the data are limited ^19, 20^. TXA also passes into breast milk at a concentration of approximately one hundredth of the concentration in the maternal blood ^21^. There was no evidence of an increased risk of death or thromboembolic events observed amongst babies breast-fed by mothers included in the WOMAN trial ^4^. However, it is important that the risk of potential harm to neonates is also assessed and considered when making recommendations about the use of TXA for preventing severe postpartum bleeding.

### Rationale for an individual patient data meta-analysis

Many randomised trials have assessed the effects of TXA in women after childbirth. Standard meta-analyses of these trials are limited to analysis of group-level ( *i.e.* aggregate) data usually extracted from the published reports. Such analyses can give more precise estimates of the effects of TXA on bleeding and thrombosis. However, they do not allow the detailed analyses of how treatment effects vary by patient characteristics that are needed to decide which patients should be treated ^22^. Techniques such as subgroup analysis and meta-regression can be used to explore if treatment effects vary by specific patient or treatment characteristics, but because they are based on aggregate data, they lack statistical power and are prone to bias, which impacts on the credibility of the results ^23, 24^. For example, a meta-regression could be used to examine how the effects of TXA vary by the average haemoglobin (Hb) of the women included in each trial. However, unless there is wide variation in the average Hb level across trials, the analysis will lack statistical power to detect a variation in treatment effect. Furthermore, such analysis is prone to bias since it involves making inferences about individuals based on group-level information. There could be important differences in patient-level haemoglobin estimates within trials that are concealed in the group-level averages. Conclusions based on trial-level average Hb data might, therefore, not reflect the true association at the patient-level. Individual patient data (IPD) meta-analyses can overcome these limitations and allow more valid estimation of how an effect of a treatment varies between groups of patients than would be achieved using aggregate data alone.

IPD also allows for the standardisation of outcome measures. This is important for our analysis since postpartum bleeding is measured and defined differently across trials. For example, some trials assess the effect on TXA on a diagnosis of postpartum haemorrhage that is based on the amount of measured or estimated blood loss, usually 500mL for vaginal birth and 1000mL for Caesarean section, although this varies considerably ^20^. Other trials define postpartum haemorrhage according to the treating clinicians’ judgements and need for additional interventions, rather than on an arbitrary level of blood loss. IPD allows the creation of new outcome measures that are common to all trials, thus increasing statistical power and reducing heterogeneity.

We will conduct a systematic review and IPD meta-analysis to determine the effectiveness and safety of TXA for preventing postpartum bleeding and to explore how the effects vary by underlying risk and other patient characteristics, to inform the decision of who to treat. 

### Objectives

To conduct a systematic review and IPD meta-analysis to quantify the effects of TXA when used for the prevention of PPH in all women giving birth. We will:

- quantify the effects of TXA on the risk of severe postpartum bleeding, thromboembolic events and other outcomes that matter to women including neonatal outcomes;
- explore whether the effectiveness and safety of TXA varies by the presence of risk factors for bleeding or thrombosis, the type of birth, maternal anaemia and timing of TXA administration, to help identify which group(s) of women are likely to receive a net benefit from TXA.

## Methods

This protocol is registered on PROSPERO (CRD42022345775).

### Ethical approval

Institutional review board (IRB) approval for this study is not required. This project involves the analysis of existing trial data. Each trial providing individual patient data will have received local ethical approval. The planned study will not require further recruitment or data collection from patients, and the analysis will not include identifiable data.

### Trial eligibility criteria

We will conduct a systematic review and IPD meta-analysis of randomised, placebo-controlled trials with 500 patients or more that assessed the effects of TXA in women giving birth vaginally or by Caesarean section. To be included, a randomised trial must: i) be prospectively registered ( *i.e.* before the first participant is enrolled) in a trial registry; randomise 500 or more patients; and have a low risk of bias arising from the randomisation process (random sequence generation and allocation concealment). Due to potential for subversion of the randomisation process ^25^, we will judge trials using sealed envelopes as a method of allocation to be at high risk of bias for allocation concealment and will not be eligible for inclusion. All eligible trials will be included irrespective of language or publication status.

### Outcomes

We referred to the core outcome sets for the prevention and treatment of postpartum haemorrhage when selecting outcomes for this review ^26^.

We will assess the effect of TXA on the following primary outcomes:

- Life-threatening postpartum bleeding. A composite outcome defined as death or surgical intervention for bleeding (laparotomy, embolization, uterine compression sutures, or arterial ligation) within 24 hours after birth.
- Fatal or non-fatal thromboembolic event. One or more of any of the following; myocardial infarction, stroke, deep vein thrombosis or pulmonary embolism as diagnosed by each trial up to the end of follow-up for each trial.

We will assess the effect of TXA on the following secondary outcomes.

***Maternal outcomes***

- Clinically significant postpartum bleeding within 24 hours after birth. Defined as bleeding after birth that leads to one or more of the following interventions for bleeding: additional uterotonics, non-trial TXA, perineal or vaginal packing, manual removal of placenta, uterine tamponade, bimanual compression, external aortic compression, non-pneumatic anti-shock garment, uterine compression sutures, arterial ligation, or arterial embolization. This outcome will be restricted to events occurring in trials involving women without severe postpartum bleeding at baseline.
- Death within 24 hours after birth.
- Death due to bleeding up to the end of follow-up for each trial.
- Haemorrhagic shock (shock index ≥1.4) within 24 hours after birth. Based on lowest recorded SBP and the associated heart rate measurement.
- Surgical intervention for bleeding (laparotomy, embolization, uterine compression sutures, or arterial ligation) within 24 hours after birth.
- Fatal or non-fatal myocardial infarction up to end of follow-up for each trial.
- Fatal or non-fatal stroke up to end of follow-up for each trial.
- Fatal or non-fatal deep vein thrombosis up to end of follow-up for each trial.
- Fatal or non-fatal pulmonary embolism up to end of follow-up for each trial.
- Hysterectomy for bleeding within 24 hours of birth. The analysis for this outcome will be stratified depending on whether or not the women have significant postpartum bleeding at baseline. Observations from trials involving women with severe postpartum bleeding at baseline suggest that the decision to perform a hysterectomy is often made prior to randomisation. Since such events cannot be influenced by the use of TXA, we do not expect to observe a treatment effect in these women. However, this may not be the case for women without postpartum bleeding at baseline, thus a stratified analysis will be conducted.
- Peripartum Hb change. Difference between last available measure before birth and the last measure taken before discharge. Estimates will be corrected for receipt of blood transfusion using coefficients from a predictive model of average Hb increment derived from a US cohort study of 23,194 hospital patients who received one unit of red blood cells, adjusted for potential effect modification by baseline Hb, BMI and age ^27^.
- Additional uterotonics within 24 hours after birth. This outcome will be restricted to events occurring in trials involving women without severe postpartum bleeding at baseline.
- Receipt of blood transfusion up to 42 days after randomisation, death or at discharge from hospital, whichever occurs first. Although there is evidence that TXA reduces blood transfusion during elective surgery ^10^, we do not anticipate that we will observe a marked reduction in blood transfusion associated with TXA in our analysis. Most of the transfusion events are likely to occur in women with anaemia or in women with severe bleeding at baseline. Although these transfusions may occur after randomisation, for many women the decision to transfuse will have been made prior to randomisation, thus could not be influenced by the use of TXA.
- Transfer to higher level of care up to 42 days after birth, death or at discharge from hospital, whichever occurs first.
- Sepsis to end of follow-up for each trial.
- Maternal quality of life including physiological, social and emotional changes measured at end of follow-up for each trial.

***Neonatal outcomes***

- Death or thrombotic events in babies exposed to the trial treatment via breast milk to end of follow-up for each trial.
- Death or thrombotic events in babies exposed to the trial treatment via placental transfer to end of follow-up for each trial. This outcome will be restricted to events occurring in trials involving the administration of TXA prior to cord clamping.
- Breastfeeding after randomisation to the end of follow-up for each trial.

### Searching for trials

Because this review will be restricted to prospectively registered trials, we will focus the search for records of potentially eligible trials on the WHO’s International Trial Registry Platform. As of October 2022, this database includes records of trial registration data sets made available by 17 data providers from throughout the world.

We will search the platform using the following terms;

(“Tranexamic Acid” OR TXA OR AMCA OR AMCHA OR Amchafibrin OR Anvitoff OR Cyklokapron OR Cyclocapron OR cyklocapron OR Exacyl OR KABI 2161 OR Spotof OR t-AMCHA OR “trans-4-(Aminomethyl)cyclohexanecarboxylic Acid” OR Transamin OR Ugurol OR Lysteda OR Cyclo-F OR Amstat OR Hexacapron OR Hexakapron OR “aminomethylcyclohexanocarboxylic acid” OR amchafibrin OR amikapron OR Amicar OR “Aminocaproic Acid” OR Afibrin OR Amica OR acikaprin OR caprogel OR Capralense OR Capramol OR Caproamin OR Caprocid OR Caprolest OR caprolisine OR CY 116 OR CY-116 OR CY116 OR ekaprol OR Epsamon OR Epsikapron OR Epsicapron OR epsilcapramin OR Hemocaprol OR Hexalense) AND (postpartum OR PPH OR post-partum OR birth OR childbirth OR caesarean OR delivery OR cesarean)

We will also check records included in the register of anti-fibrinolytic trials maintained by the LSHTM CTU’s Global Health Trials Group, as well as check reference lists of relevant articles, and correspond with trialists to identify any further trials. The searches will not be restricted by language or publication status.

### Selecting trials

The output from the WHO ICTRP will be exported as a CSV file and opened in Microsoft Excel. One review team member will examine the records to identify potentially eligible trials. The full texts of these potentially eligible trial reports will be retrieved and assessed against the inclusion criteria. Two review team members will independently extract information on trial characteristics, methods, and aggregate outcome data using an extraction form. Disagreements will be resolved through discussion or after consultation with a third review team member if required.

### Assessing risk of bias

We will use a modified version of Cochrane’s risk of bias tool to address the following questions for each outcome of interest for each trial ^28^;

•   Was the allocation sequence adequately generated?

•   Was the allocation adequately concealed?

•   Was knowledge of the allocated interventions adequately prevented?

•   Was loss to follow-up (missing outcome data) infrequent?

•   Was the trial apparently free of other problems that could put it at risk of bias?

We will extract information and respond to each question as ‘definitely yes’ (low risk of bias), ‘probably yes’, ‘probably no’, or ‘definitely no’ (high risk of bias). Two members of the review team will independently assess the risk of bias in each included trial. We will resolve disagreements through discussion and with involvement of a third team member if required.

### Collecting individual patient data

We will follow a similar approach to that described previously by the Anti-fibrinolytics Trialists Collaboration ^29^. We will contact the named investigator (as specified in the final trial publication or the trial registration record) for each trial and provide them with the IPD meta-analysis protocol and a cover letter explaining what the study is about. If we receive no response from the named investigator, we will contact another trial investigator. The investigators of the eligible trials will be invited to join the Trialists Collaboration and we will request the anonymised, IPD from all eligible trials.

### Confidentiality, data storage and handling

We will again follow a similar approach to that described previously by the Anti-fibrinolytics Trialists Collaboration ^29^. All IPD data supplied to the project team will be held securely at the LSHTM CTU Global Health Trials Group in adherence to all relevant legislation, guidelines, and regulatory requirements. The data will be used for the purposes of medical research only and within the constraints of consent under which the data were provided. Supplied data will not be shared with others outside of the project group without the permission of the responsible trialist. No individual patients will be identified in any publications or presentations prepared by the collaborative group.

Data received will be stored on a secure server within an ISO 27001-compliant data centre. Data will only be accessible by the project team and authorised personnel. Electronic data will be protected by any or all of the following: assigned logins, protected network areas and encryption. 

We will check the IPD for consistency and completeness. We will compare baseline data with estimates reported in the trial publications and refer any queries back to the responsible trialists for clarification. 

Trialists will be able to withdraw their IPD from the analyses at any time. 

### Data analysis

We will use a one-stage model to analyse the data for each outcome. This approach combines IPD in a single meta-analysis based on a regression model stratified by trial and allows for the investigation of within- and between-trial variances, as well as estimation of the treatment effect in a single analytical model. We will include data on all randomised women on an intention-to-treat basis.

For binary outcomes we will report results as odds ratios and 95% confidence intervals and for continuous outcomes we will report mean differences and 95% confidence intervals.

First, we will assess the homogeneity of the treatment effects between trials by estimating a random effects model in which the intercept and the treatment effect will be allowed to have a distribution across trials. The variance of the distribution of the treatment effect will indicate the heterogeneity between trials. If, however, only a small number of trials are included, we will instead examine the heterogeneity by including an interaction term between the treatment and the trial variable and report the p-value. We will consider a p-value <0.05 to indicate statistical heterogeneity. 

***Subgroup analyses***

*Risk: Does the effectiveness and safety of TXA depend on underlying risk?*

Understanding whether and how the effects of TXA on significant postpartum bleeding and thromboembolic events vary by the underlying risks of these outcomes will help to identify the women for whom the potential benefit of TXA outweighs the potential harm. To explore this, we will develop prognostic models to estimate the underlying risks of life-threatening bleeding, clinically significant postpartum haemorrhage, and thromboembolic events using IPD from the included trials. We will only use baseline characteristics collected before randomisation as potential predictors and will use data from both the treatment and placebo groups to improve precision. We will use the backward stepwise method and remove, one at a time, variables for which there is no evidence of association (p-value for the Wald test >0.05). The predicted underlying risk for all outcomes will be estimated for each trial participant after adjusting for the use of TXA. We will assess the performance of the models by estimating discrimination and calibration. Using the models, data for each woman obtained from the included trials will be assigned to a category of risk depending on the distribution of the outcomes. We will calculate effect estimates within each category which we will examine for statistical evidence of heterogeneity ( *i.e.* p<0.001). Previous analyses of the effects of TXA in severely bleeding patients suggest that the relative effects of TXA do not vary by the underlying risk of death in these patients ^30^. Based on this evidence, we do not anticipate statistical heterogeneity in the effects of TXA by the underlying risks of life-threatening bleeding, clinically significant postpartum haemorrhage, or thromboembolic events in our analysis.

*Maternal anaemia: Does the effectiveness and safety of TXA depend on the severity of anaemia?*

Although severely anaemic women have a much higher risk of postpartum haemorrhage ^13^, we expect that TXA will reduce the risk of life-threatening haemorrhage and clinically significant postpartum bleeding by a similar proportion, regardless of the severity of anaemia. Similarly, we do not expect the effect of TXA on risk of thromboembolic events to differ by severity of anaemia.

There is evidence to suggest that fibrinolysis is worse in women with anaemia, which might suggest that TXA would have a greater effect on the risk life-threatening bleeding and that any increased risk of thromboembolic events would be less in women with lower baseline Hb levels ^31^. However, recent results from *in vitro* studies do not support this hypothesis; rather, they suggest that red blood cells may increase the potency of TXA ^32^. Furthermore, the POISE-3 trial of TXA in non-orthopaedic surgery which observed a reduction in the risk of major bleeding with TXA, found no evidence of heterogeneity according to pre-operative Hb level in a prespecified subgroup analysis ^11^. To explore this, we will assess the impact of baseline maternal Hb on the effects of TXA in a regression analysis that includes continuous terms for maternal Hb and its square and their interaction with treatment. We will consider a p-value <0.001 as evidence for the presence of statistical heterogeneity.

*Type of birth: Does the effectiveness and safety of TXA depend on the type of birth?*

We do not anticipate the effects of TXA to vary by type of birth (vaginal or Caesarean section). Indeed, the WOMAN trial of TXA use in women with established severe postpartum bleeding which observed a reduction in the risk of death due to bleeding, found no evidence that the effect differed by type of birth ^4^. However, because Caesarean section is an established risk factor for severe bleeding and thromboembolic events after birth, we will examine whether the effects of TXA on these outcomes vary between vaginal birth and Caesarean section. We will calculate effect estimates for each type of birth (vaginal birth and Caesarean section) which will be examined for statistical evidence of heterogeneity ( *i.e.* p<0.001).

*Presence of clinically significant postpartum bleeding at baseline: Does the effectiveness and safety of TXA depend on whether it is given before or after the onset of clinically significant postpartum bleeding?*

There is strong evidence that the effect of TXA on the risk of death in patients with severe bleeding varies by the timing of treatment, with early treatment being the most effective ^33^. This raises the possibility that giving TXA immediately before or after birth, and before the onset of severe postpartum bleeding, may have a greater effect on the risk of life-threatening bleeding than when it is given to women with established bleeding. We therefore expect that the effects of TXA on life-threatening bleeding in our analysis will vary depending on whether TXA is given before or after the onset of severe bleeding, with administration of TXA before or immediately after birth to prevent severe bleeding being more effective than administration of TXA to treat established severe bleeding. Since there is no evidence that the effect of TXA on risk of thromboembolic events varies by timing of treatment ^33^, we do not expect that the effect of TXA on the risk of thromboembolic events will similarly vary. We will examine these hypotheses by conducting analyses of the effects of TXA on life-threatening bleeding and thromboembolic events according to whether TXA is given before or after the onset of severe postpartum bleeding. We will calculate effect estimates for each group (TXA given before or after onset of clinically significant postpartum bleeding) which we will examine for statistical evidence of heterogeneity ( *i.e.* p<0.001)

We will assess the credibility of any observed subgroup effects using the Instrument to Assess the Credibility of Effect Modification Analyses (ICEMAN) tool ^24^.

### Unit of analysis issues

For the neonatal outcomes we will use generalised linear mixed models, with a maternal level random treatment effect, to take into account multiple births.

### Sensitivity analysis

It is possible that IPD will not be available from all eligible trials. In this event, we will describe any differences between the characteristics of trials contributing IPD and those for which IPD are not available. Where possible, we will also conduct sensitivity analyses incorporating the available aggregate data to explore the robustness of results based on IPD alone.

### Summary of findings and assessment of the certainty of the evidence

We will follow the methods described in the Cochrane Handbook for Systematic Reviews of Interventions ^34^, and use the MAGICapp platform ^35^ to present the main results in summary of findings tables. We will include the following outcomes:

- Life-threatening bleeding within 24 hours after birth
- Clinically significant postpartum bleeding within 24 hours after birth
- Fatal or non-fatal thromboembolic event to end of follow-up of each trial
- Death within 24 hours after birth
- Haemorrhagic shock (shock index ≥1.4) within 24 hours after birth
- Surgical interventions for bleeding within 24 hours after birth
- Peripartum Hb change (difference between last available measure before birth and the last measure taken before discharge)

We will produce a summary of findings table for each category of risk, for each effect modifier that is judged to be credible according to the ICEMAN tool ^24^.

We will follow the GRADE approach to assess the certainty of the evidence by considering the following for each outcome:

- Impact of risk of bias of individual trials;
- Precision of pooled estimate;
- Inconsistency or heterogeneity (clinical, methodological and statistical);
- Indirectness of evidence;
- Impact of selective reporting and publication bias on effect estimate.

We will rate the certainty of the evidence for each outcome as follows;

- High: we are very confident that the true effect lies close to that of the estimate of the effect.
- Moderate: we are moderately confident in the effect estimate; the true effect is likely to be close to the estimate of the effect, but there is a possibility that it is substantially different.
- Low: our confidence in the effect estimate is limited; the true effect may be substantially different from the estimate of the effect.
- Very low: we have very little confidence in the effect estimate; the true effect is likely to be substantially different from the estimate of effect.

We will use a minimally contextualised approach by which no difference between groups (e.g. odds ratio of 1.0) will be taken as the threshold for rating the certainty.

### Amendments

New evidence will be incorporated as it becomes available and new hypotheses may emerge. Any consequent protocol amendments will be detailed in a revised protocol document that will be dated and assigned a new version number.

## Patient and public involvement

Patient and public representatives were not involved in the design of this IPD analysis. 

## Data Availability

No data are associated with this article. This protocol has been reported in line with current PRISMA-P guidelines ( 10.6084/m9.figshare.21388326).
