# Supplementary material for: Tranexamic acid for the prevention of postpartum bleeding: Protocol for a systematic review and individual patient data meta-analysis
Source: Gates Open Res. 2023 May 12;7:3. Originally published 2023 Jan 19. [Version 2] doi: 10.12688/gatesopenres.13747.2 (PMC10439279; doi:10.12688/gatesopenres.13747.2)
Supplement: Peer review history

# Peer review: Reviewer response for version 2

**DOI:** 10.21956/gatesopenres.16000.r34287

**Published:** 2023-08-18

**Authors:** Zahida Qureshi, Alfred Osoti

**Affiliations:** 1 University of Nairobi, Nairobi, Nairobi County, Kenya

Thank you for giving us the opportunity to review this this well-written protocol "Tranexamic acid for the prevention of postpartum bleeding: Protocol for a systematic review and individual patient data meta-analysis"

 The response to the previous reviewers comments have improved the quality of the protocol and we do not have any substantive comment to add.

 We do however wish to re-address the point of restricting the inclusion of RCT's involving 500 or more patients. We have noted the response to this comment, but feel that the authors may wish to re-look at cut-off.

 We have added one recent citation which the authors may consider including (Cheema *et al.*, 2023 ^1^).

 Overall this IPD meta-analysis is comprehensive in the maternal, newborn outcomes and public and patient factors that will be analysed.

Is the study design appropriate for the research question?

Yes

Is the rationale for, and objectives of, the study clearly described?

Yes

Are sufficient details of the methods provided to allow replication by others?

Yes

Are the datasets clearly presented in a useable and accessible format?

Not applicable

Reviewer Expertise:

Randomized controlled trials, maternal and new born health

We confirm that we have read this submission and believe that we have an appropriate level of expertise to confirm that it is of an acceptable scientific standard.

## References

[1] : Tranexamic acid for the prevention of blood loss after cesarean section: an updated systematic review and meta-analysis of randomized controlled trials. *Am J Obstet Gynecol MFM* .2023;5(8) : 10.1016/j.ajogmf.2023.101049 101049 10.1016/j.ajogmf.2023.101049 37311484

# Peer review: Reviewer response for version 1

**DOI:** 10.21956/gatesopenres.15036.r32860

**Published:** 2023-02-24

**Authors:** Pollyanna Hardy

**Affiliations:** 1 National Perinatal Epidemiology Unit, Clinical Trials Unit, University of Oxford, Oxford, UK

This article presents the protocol for a systematic review and an independent patient data meta-analysis (IPD) for the use of tranexamic acid (TXA) to prevent postpartum bleeding. TXA has previously been shown to be effective for the treatment of severe postpartum bleeding. This systematic review and IPD will provide valuable evidence for its indication for all women immediately prior to and after birth, importantly addressing the potential benefits and harms to women and their babies, and includes relevant subgroup analyses to explore which women may benefit.

 The research is well justified and the protocol is clearly written with appropriate methodology.

 I have the following comments that I think would improve the paper further.

1. It should be made clear from the outset what the target population is and whether the research question is addressing prophylactic use of TXA in all birthing women, or whether this is preventative treatment for women at risk of post-partum bleeding.
2. Potential harms to the baby are not discussed or mentioned in the introduction or objectives (via placental transfer or breast milk), although they are included as secondary outcomes. This should be addressed to provide a background as to why neonatal outcomes are important to include.
3. Why are only studies with 500 patients or more included, and what impact will this have on the risk of publication bias?
4. It’s not clear what type of outcome the second primary outcome is: Fatal and non-fatal thromboembolic events. Is this a binary composite outcome (i.e. at least one thromboembolic event), or a continuous outcome (i.e., number of thromboembolic events)?
5. Will the neonatal outcome relating to TXA exposure via placental transfer be restricted to events occurring in trials involving women treated prior to birth only? (i.e., excluding trials where randomisation occurred postpartum).
6. In the analysis section, please clarify which measure of the treatment effect will be used for the continuous outcomes (currently only odds ratios are stated).
7. For the neonatal outcomes, how will the analysis allow for the clustering effect of multiple births?
8. Could the authors please comment on the involvement of patients and the public in the various stages of this work?

Minor comments:

- PROSPERO details do not currently correspond with the protocol submission, especially the listed outcomes. These should be aligned.
- In the section on “Why this research is needed”, the statement “We need to be more cautious about offering interventions to patients at low underlying risk since the balance of risks and benefits is more uncertain in these patients.” I assume this is in comparison to patients with a high underlying risk. This should be clarified.
- The second objective does not currently fully correspond with the listed subgroup analyses: timing of when TXA is given is currently not reflected in the objectives statement.
- It would be helpful to the reader to use subtitles within the secondary outcomes section to identify ‘Maternal’ and ‘Neonatal’ outcomes.

Is the study design appropriate for the research question?

Yes

Is the rationale for, and objectives of, the study clearly described?

Yes

Are sufficient details of the methods provided to allow replication by others?

Yes

Are the datasets clearly presented in a useable and accessible format?

Not applicable

Reviewer Expertise:

Randomised controlled trials, statistics and methodology, pregnancy and the newborn

I confirm that I have read this submission and believe that I have an appropriate level of expertise to confirm that it is of an acceptable scientific standard.

# Response

**Published:** 2023-04-24

**Authors:** Katharine Ker

**Affiliations:** London School of Hygiene & Tropical Medicine, UK

The research is well justified and the protocol is clearly written with appropriate methodology. 

**Thank you. We appreciate this positive feedback on our article.**

 I have the following comments that I think would improve the paper further. 

 1. It should be made clear from the outset what the target population is and whether the research question is addressing prophylactic use of TXA in all birthing women, or whether this is preventative treatment for women at risk of post-partum bleeding. 

**The target population is all women giving birth irrespective of their risk of severe postpartum bleeding. We have added further detail to the Abstract and the Objectives sections in the revised article to clarify this.**

 2. Potential harms to the baby are not discussed or mentioned in the introduction or objectives (via placental transfer or breast milk), although they are included as secondary outcomes. This should be addressed to provide a background as to why neonatal outcomes are important to include. 

**We agree that this should be added. We have added details on potential neonatal harms to the ‘Why this research is needed’ section of the Background and have added this to the Objectives in the revised article.** 

 3. Why are only studies with 500 patients or more included, and what impact will this have on the risk of publication bias? 

**The decision to restrict trials to those including 500 patients or more is in accordance with the inclusion criteria agreed by all members of the Anti-fibrinolytic Trialists Collaboration and is detailed in the overarching protocol of ATC (available at https://txacentral.lshtm.ac.uk/wp-content/uploads/2021/09/Protocol_12-April-19.pdf).**

**We have previously systematically reviewed trials assessing the effects of TXA including those assessing the use of TXA for preventing PPH ( https://obgyn.onlinelibrary.wiley.com/doi/10.1111/1471-0528.14267). The majority of these trials are small (<100 patients) and poor quality, and there are serious concerns about the integrity of the data from a proportion of these trials. The inclusion of these trials requires much more work to obtain and check the data than is required for larger trials which are typically better reported and more reliable. We judge that the amount of work and resources required to include IPD from small trials is not justified when we consider the minimal (often zero) contribution they make to the relative effect estimates.**

**Regarding the impact on the risk of publication bias, we believe that this will be minimal. In fact, publication bias is more likely to arise with the inclusion of small trials, since small trials showing non-statistically significant differences are less likely to be published than larger trials which tend to be published regardless of the results. Our decision to focus on larger trials (i.e. involving ≥500 patients) will improve the reliability of the results by avoiding bias (such as publication bias) resulting from small study effects.**

 4. It’s not clear what type of outcome the second primary outcome is: Fatal and non-fatal thromboembolic events. Is this a binary composite outcome (i.e. at least one thromboembolic event), or a continuous outcome (i.e., number of thromboembolic events)? 

**The second primary outcome ‘fatal and non-fatal thromboembolic events’ is a binary outcome with each event representing one patient experiencing one or more thromboembolic event.  We have amended the wording of this outcome in the revised article to clarify this.**

****

 5. Will the neonatal outcome relating to TXA exposure via placental transfer be restricted to events occurring in trials involving women treated prior to birth only? (i.e., excluding trials where randomisation occurred postpartum). 

**Yes, this outcome will be restricted to data from trials involving women receiving TXA before cord clamping. We have added further detail to this outcome in the revised article to clarify this.**

****

 6. In the analysis section, please clarify which measure of the treatment effect will be used for the continuous outcomes (currently only odds ratios are stated). 

**Thank you to pointing out this omission. Continuous outcome data will be reported as mean differences and 95% confidence intervals. This has been added to the ‘Data analysis’ section in the revised article.** 

 7. For the neonatal outcomes, how will the analysis allow for the clustering effect of multiple births? 

**We will use generalised linear mixed models, with a maternal level random treatment effect, to take into account multiple births. We have added this detail under the subheading ‘Unit of analysis issues’ within the ‘Data analysis’ section of the revised article.** 

 8. Could the authors please comment on the involvement of patients and the public in the various stages of this work? 

**Patient and public representatives were not involved in the design of this IPD analysis. We have added text to confirm this under the ‘Patient and public involvement’ subheading of the revised article. ** 

 Minor comments: 

 • PROSPERO details do not currently correspond with the protocol submission, especially the listed outcomes. These should be aligned. 

**We have reviewed the details in the PROSPERO record and made edits to this record to ensure the outcomes correspond. However, due to word constraints imposed by the PROSPERO system, the outcomes are described in less detail in the PROSPERO record than in this article.**

 • In the section on “Why this research is needed”, the statement “We need to be more cautious about offering interventions to patients at low underlying risk since the balance of risks and benefits is more uncertain in these patients.” I assume this is in comparison to patients with a high underlying risk. This should be clarified. 

**Yes, the reviewer is correct. We have revised this statement in the revised article to make this clearer.**

 • The second objective does not currently fully correspond with the listed subgroup analyses: timing of when TXA is given is currently not reflected in the objectives statement. 

**Thank you for pointing out this omission. We have corrected this by adding ‘timing of TXA administration’ to the second objective**. 

 • It would be helpful to the reader to use subtitles within the secondary outcomes section to identify ‘Maternal’ and ‘Neonatal’ outcomes. 

**We agree and have added subheadings to distinguish maternal and neonatal outcomes in the list of secondary outcomes**.
